# Chemopreventive effect of chalcone derivative, L2H17, in colon cancer development

**DOI:** 10.1186/s12885-015-1901-x

**Published:** 2015-11-09

**Authors:** Shanmei Xu, Minxiao Chen, Wenbo Chen, Junguo Hui, Jiansong Ji, Shuping Hu, Jianmin Zhou, Yi Wang, Guang Liang

**Affiliations:** 1 Chemical Biology Research Center, School of Pharmaceutical Sciences, Wenzhou Medical University, Wenzhou, 325035 China; 2 Department of Radiology, the 5th Affiliated Hospital, Wenzhou Medical University, Lishui, Zhejiang China

**Keywords:** Colon cancer, Chalcone derivatives, Metastasis, NF-κB, Akt

## Abstract

**Background:**

Colon cancer is the third most commonly diagnosed cancer and the second leading cause of cancer mortality worldwide. Chalcone and its derivatives are reported to exhibit anti-cancer effects in several cancer cell lines, including colon cancer cells. In addition, chalcones have advantages such as poor interaction with DNA and low risk of mutagenesity. In our previous study, a group of chalcone derivatives were synthesized and exhibited strong anti-inflammatory activities. In this study, we evaluated the anti-cancer effects of the chalcone derivative, L2H17, in colon cancer cells.

**Methods:**

The cytotoxicities of L2H17 on various colon cancer cell lines were investigated by MTT and clonogenic assay. Cell cycle and apoptosis analysis were performed to evaluate the molecular mechanism of L2H17-mediated inhibition of tumor growth. Also, scratch wound and matrigel invasion experiments were performed to estimate the cell migration and invasion after L2H17 treatment. Finally, we observed the anti-colon cancer effects of L2H17 in vivo.

**Results:**

Our data show that compound L2H17 exhibited selective cytotoxic effect on colon cancer cells, via inducing G0/G1 cell cycle arrest and apoptosis in CT26.WT cells. Furthermore, L2H17 treatment decreased cell migration and invasion of CT26.WT cells. In addition, L2H17 possessed marked anti-tumor activity in vivo. The molecular mechanism of L2H17-mediated inhibition of tumor promotion and progression were function through inactivated NF-κB and Akt signaling pathways.

**Conclusions:**

All these findings show that L2H17 might be a potential growth inhibitory chalcones derivative for colon cancer cells.

## Background

Colon cancer is the third most commonly diagnosed cancer and the second leading cause of cancer mortality worldwide [1]. Although the mortality rate is decreasing as the result of improved detection, the incidence of colon cancer continues to increase [2]. The development of colon cancer is a complicated process, including complex interactions among genetic alterations, environmental carcinogens, and host immune system [3]. There is increasing evidence that chronic inflammation is an important pathogenic factor in the initiation and progression of colon cancer. For example, inflammatory bowel disease is an important risk factor for the development of colon cancer [4].

Chalcone is an important secondary plant metabolite which shows an array of pharmacological properties [5]. In addition, chalcones have been reported to exhibit several biological activities, including anti-tumor, anti-inflammatory, immunomodulatory, anti-bacterial, anti-malarial, anti-leishmanial, trypanocidal and nitric oxide inhibitory activity [6, 7]. Synthetic analogs and derivatives of the molecule are increasingly getting into focus due to their promising therapeutic potential. Chalcone derivatives are reported to exhibit anti-cancer effects in several cancer cell lines, such as human acute leukemia cells [8], colon cancer cells [9, 10], human bladder cancer cells [11], and lung cancer cells [12]. In addition, as anticancer therapeutic agents, chalcones have advantages such as poor interaction with DNA and low risk of mutagenesity [13].

**Fig. 1:**
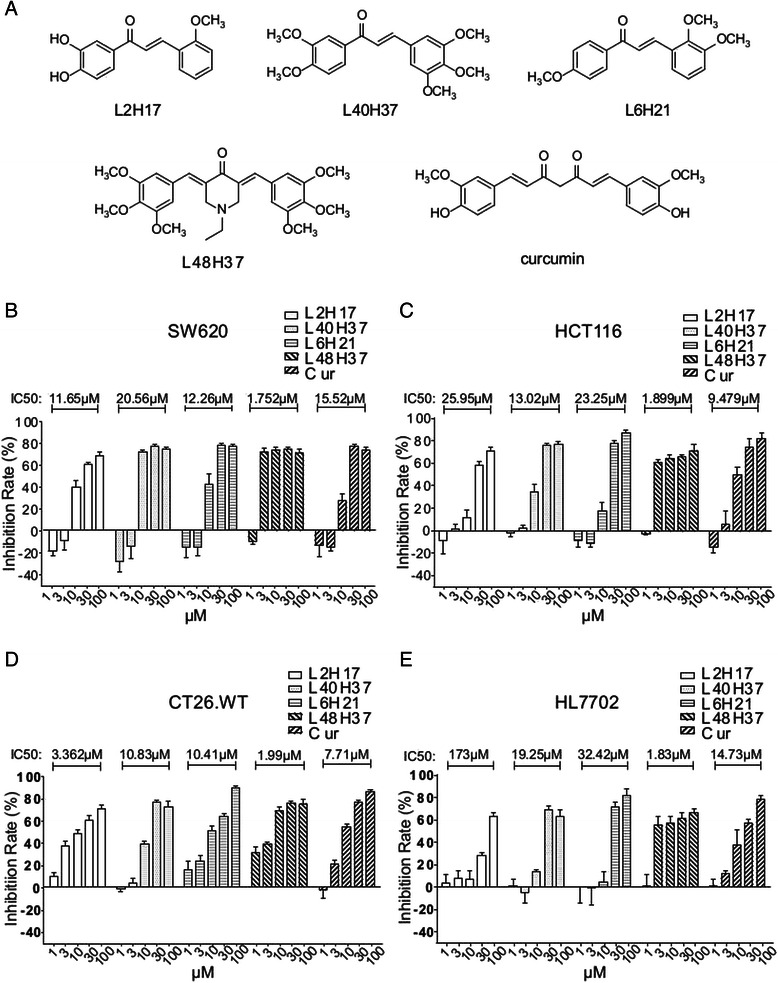
Chalcone derivatives reduced viability of colon cancer cells. (**a**) Structures of curcumin and chalcone derivatives. Colon cancer cell lines, such as SW620 (**b**), HCT116 (**c**), CT26.WT (**d**), and human hepatocytes HL-7702 cells (**e**) were treated with chalcone derivatives (L2H17, L40H37, L6H21, or L48H37) or curcumin at various concentrations (1, 3, 10, 30, or 100 μM) for 48 h, while DMSO was used as the vehicle control. After 48-h treatment, the cell proliferation of each group was assessed by MTT assay. The data were obtained from three independent experiments performed in triplicate, and the results were expressed as percentage of vehicle (DMSO) control. The data were presented as mean ± SEMs, and fitted with GraphPad Prism 5.0 to obtain the IC_50_ values

In our previous study, a group of chalcone derivatives were synthesized and screened for anti-inflammatory activities. Some of the compounds, including L2H17, L40H37, L6H21, and L48H37 exhibited excellent anti-inflammatory effects in LPS-stimulated macrophages (structures of these chalcone derivatives are shown in Fig. 1a) [14]. A lot of studies have revealed that there is an association between inflammation and cancer development [15]. Thus, we hypothesized that these chalcone derivatives may exhibit anti-cancer effect in colon cancer cells. In the present study, we show that L2H17 possesses the best anti-tumor effect on colon cancer cell lines and lowest cytotoxicity on noncancerous cell among these four compounds. Further biological experiments of L2H17 on its anti-tumor effects *in vitro* and in vivo, the molecular mechanisms of L2H17-mediated inhibition of tumor promotion and tumor progression were then examined. The findings obtained here may help us to develop novel plant-derived chalcones used for potent chemotherapeutic agents against colon cancer.

## Methods

### Cell lines and reagents

SW620 (human colon cancer cell line), HCT 116 (human colon cancer cell line), CT26.WT (mouse colon cancer cell line), and HL-7702 (human hepatocytes) were obtained from Shanghai Institute of Biosciences and Cell Resources Center (*Chinese Academy of Sciences, Shanghai, China*) and cultured in RPMI-1640 or DMEM (*Gibco/BRL life Technologies, Eggenstein, Germany*), with 10 % heat-inactivated FBS (*Gibco/BRL life Technologies, Eggenstein, Germany*), 1 % penicillin/streptomycin solution (*Mediatech Inc., Manassas, VA*) in a humidified atmosphere of 5 % CO_2_ at 37 °C. Antibodies for IκB-α, cleaved PARP-1, Akt1/2, Bax, Bcl-2, procaspase-3, E-cadherin, lamin B, goat anti-rabbit IgG-HRP, mouse anti-goat IgG-HRP, and donkey anti-goat IgG-HRP were obtained from Santa Cruz Biotechnology (*Santa Cruz, CA*); and antibodies for GAPDH, cleavaged caspase-3 and p-Akt were obtained from Cell Signaling Technology (*Danvers, MA*). FITC Annexin V apoptosis Detection Kit I was purchased from BD Pharmingen (*Franklin Lakes, NJ*). Matrigel was from BD Biosciences (*Shanghai, China*). Mitomycin C was purchased from Sigma (*Louis, MO*). Chalcone derivatives were synthesized and characterized as described in our previous publication [14]. Compounds with a purity of 98.9 %, were dissolved in DMSO for *in vitro* experiments, and were dissolved in 1 % sodium carboxyl methyl cellulose-Na (CMC-Na) for in vivo experiments.

### Animals

Female BALB/c mice weighing 18–20 g were obtained from the Animal Center of Wenzhou Medical University (*Wenzhou, China*). Animals were housed at a constant room temperature with a 12:12 h light-dark cycle, and fed with a standard rodent diet and water. The animals were acclimatized to the laboratory for at least 3 days before used. Protocols involving the use of animals were approved by the Wenzhou Medical University Animal Policy and Welfare Committee (Approval documents: wydw2014-0062).

### MTT assay

Cells (5 × 10^3^ cells/well) were seeded in 96-well plates for 24 h and then treated with chalcone derivatives or curcumin with different doses (1, 3, 10, 30 or 100 μM) for 48 h, while DMSO was used as the vehicle control. After treatment, a fresh solution of MTT (5 mg/mL) prepared in phosphate buffer solution (PBS) was added to the cells in each well for 4 h at 37 °C. Then the MTT was aspirated, and the formazan crystals resulting from mitochondrial enzymatic activity on the MTT substrate were dissolved with 150 μL of DMSO; absorbance at 490 nm was recorded by the multiwell-plate reader at 490 nm. Each experiment was done in triplicate, and repeated for three times; the mean of the three values from three independent experiments was determined, and the results were expressed as percent of the vehicle control. IC_50_ values of each compounds was calculated by GraphPad Pro 5.0 (*San Diego, CA*).

### Dynamic monitoring of CT26.WT cell proliferation using the RT-CES system

The dynamic monitoring of CT26.WT cell proliferation after treatment was performed using the ET-CES system (*Acea Biosciences Inc., San Diego, CA*). CT26.WT cells were plated at a density of 6 × 10^4^ cells/well in 300 μL of medium in 8 well E-plates and the installed plates were placed in a standard cell culture incubator, at 37 °C in a humidified atmosphere of 5 % CO_2_ overnight. After cell seeded, the integrated software was used to record and analyze the data during 0-100 h. And L2H17 or curcumin at different concentrations (0, 1, 3, 10, 30, 100 μM) was added to the medium at 24 h after incubation. The cell index represents the quantitative measure of the spreading and/or proliferative status of the cells in an electrode-containing well.

### Clonogenic assay

Cells were seeded in 6-well plates (500 cells/well) in 3 mL of RPMI-1640 medium at 37 °C in 5 % CO_2_ atmosphere overnight. L2H17 (1, 3, or 10 μM), curcumin (10 μM) or vehicle (DMSO) was added to the cells for 7 days. The culture medium was replaced by the fresh drug-containing medium every two days to keep cells growing for 7 days. Colonies were washed with PBS, fixed with 4 % methanol at room temperature for 15 min, washed with purified water for 3 times and stained with Crystal violet for 10 min. Colonies containing more than 50 cells were counted, and visualized colonies were then photographed and calculated. Each experiment was done in triplicate for three independent experiments.

### Cell cycle analysis

CT26.WT cells (5 × 10^5^ cells/well) were seeded in 6-well plates and allowed to adhere overnight. The nest day, the cells were treated with L2H17 (1, 3, 10 and 30 μM), curcumin (30 μM) or vehicle (DMSO) for 24 h. Then the cells were trypsinized, washed, and fixed in 75 % ice-cold ethanol at -20 °C overnight. After centrifugation, the pellets were washed with cold PBS, suspended in 500 μL PBS with 50 mg/ml propidium iodide (PI) and incubated at 37 °C for 40 min in the dark. The cell cycle distribution was determined using Becton Dickinson FACSCalibor (*Franklin Lakes, NJ*) and analyzed by ModFit LT (*Verity Software House, Topsham, ME*). Each experiment was done in triplicate for four independent experiments.

### Cell apoptosis analysis

CT26.WT cells (5 × 10^5^ cells/well) were seeded in 6-well plates and allowed to adhere overnight, and then treated with L2H17 (1, 3, 10 and 30 μM), curcumin (30 μM), or vehicle (DMSO) 48 h. The cells were harvested and stained with Annexin V and PI. Flow-cytometric analysis was performed using FACScalibor. All experiments were repeated for four times.

### Scratch wound model

CT26.WT cells (5 × 10^5^ cells/well) were seeded in 6-well plates and allowed to adhere overnight. At 80–90 % confluence, “reference line” was scratched at the bottom of the plate using a sterile 10 μL pipette tip. After being washed with PBS thrice, cells were further incubated with L2H17 (10 μM), curcumin (10 μM) or vehicle (DMSO) in the presence of mitomycin C (8 μg/ mL) in calcium-free RPMI-1640 to study the cell migration in the absence of cell proliferation. Photomicrographs of cells migrating across the “reference line” were taken in different fields after each treatment at 0, 24 and 48 h with a Canon digital camera (*Canon Inc., Tokyo, Japan*), respectively. The rate of mobility was quantified by the migrated distance of cells moved from the “reference lines” toward the center as compared to control. All experiments were repeated for four times.

### Matrigel invasion assay

The effects of L2H17 on invasion of CT26.WT cell were studied by a modified Boyden chamber Matrigel method using 24-well Transwell plates with 8 μm pore size (*Corning Costar Corp, Shanghai, China*) coated with 10 μL Matrigel (diluted 1:9 with serum-free medium, *BD Biosciences, Shanghai, China*). In brief, 5 × 10^5^ CT26.WT cells suspended in 200 μL serum-free RPMI 1640 culture medium were plated into the upper Matrigel-coated chamber, and then L2H17 (1, 3, or 10 μM), curcumin (1, 3, or 10 μM), or vehicle (DMSO) was loaded to the cell suspension. The bottom chamber was loaded with 500 μL of RPMI 1640 medium containing 10 % FBS, and the cells were then allowed to invade for 24 h at 37 °C. The cells that invaded into the bottom chamber were fixed in 1 % methanol for 15 min, stained with crystal violet for 25 min, and washed with PBS for three times. The images were visualized under a light microscope (×100; *Nikon, Tokyo, Japan*) and photographed. All experiments were independently repeated for four times. Total number of invaded cells was counted and presented as the ratio of the numbers of invasive cells compared to that of DMSO control.

### Western blot assay

After treated with L2H17, curcumin, or vehicle (DMSO), CT26.WT cells were harvested and lysed, supernatants were collected. The protein concentration was determined and balanced with purified water. After the sample loading buffer was added, 80 μg of protein samples were electrophoresed and then transferred to nitrocellulose membranes (*Bio-Rad Laboratories, Hercules, CA*). Each membrane was blocked at room temperature for 1.5 h and incubated with specific primary antibodies for 2 h at room temperature and at 4 °C overnight. After incubated with horseradish peroxidase-conjugated secondary antibodies for 1 h, the immunoreactive bands were visualized using enhanced chemiluminescence reagents (*Bio-Rad Laboratories, Hercules, CA*). The amounts of the proteins were analyzed using ImageJ analysis software version 1.38e (National Institutes of Health, Bethesda, MD) and normalized to their respective controls.

### Preparation of nuclear extracts

Nuclear protein extraction from CT26.WT cells were obtained by using nuclear protein extraction kit (*Beyotime Biotech, Nantong, China*) according to the manufacturer’s instructions. The nuclear extract (30 μg protein) was used for the western blot analysis.

### In vivo anti-tumor study

CT26.WT cells were harvested and injected intravenously into the BALB/c mice (3 × 10^5^ cells/mouse, in 200 μL of RPMI-1640). Simultaneously, the mice were administered orally with either L2H17 (25 mg/kg/day or 50 mg/kg/day), curcumin (50 mg/kg/day or 100 mg/kg/day), or vehicle (1 % CMC-Na) for 60 days, while the tumor-free control mice were treated with the L2H17 (50 mg/kg/day), curcumin (100 mg/kg/day), or 1 % CMC-Na as the negative control (*n* = 9 in each group). The survival and bodyweight were recorded for 60 days.

### Statistical analyses

Data are represented as mean ± SEM of three or four independent experiments. Student’s *t*-test was performed to determine statistical significance between two groups using GraphPad Prism 5.0 (*San Diego, CA*). Survival data are presented as Kaplan-Meier survival curves, and differences between groups were analyzed by the log-rank test using GraphPad Prism 5.0 (*San Diego, CA*). A *P* value < 0.05 was considered to be statistically significant.

## Results

### L2H17 shows selective cytotoxic effect on colon cancer cells

We screened the cytotoxic effect of L2H17, L40H37, L6H21, and L48H37 in both human and mouse colon cancer cells through MTT assay. Curcumin, a structure analogue of chalcone derivatives with chemopreventive effect against colon carcinogenesis, was used as the positive control in the screening [16–18]. As shown in Fig. 1, treatment of these four chalcone derivatives dose-dependently reduced cell viability in SW620 (Fig. 1b), HCT 116 (Fig. 1c), and CT26.WT (Fig. 1d), with IC_50_ values much lower than those of curcumin. Among them, L48H37 exhibited the most potent cytotoxic effect against all tested colon cancer cell lines (IC_50_ value of 1.752 μM in SW620, IC_50_ value of 1.899 μM in HCT116, and IC_50_ value of 1.99 μM in CT26.WT). An important attribute of an effective anticancer drug is cancer selectivity in its cytotoxic effect. Thus, we also screened the cytotoxic effect of these compounds against human hepatocyte cell line, HL-7702. As shown in Fig. 1e, only compound L2H17 exhibited low toxicity effect in HL7702, with an IC_50_ value of 173 μM, which is 10 times higher than that of curcumin. These results, therefore, suggest that L2H17 exhibits a certain level of colon cancer selectivity and safety in terms of its cytotoxic effect *in vitro*.

**Fig. 2:**
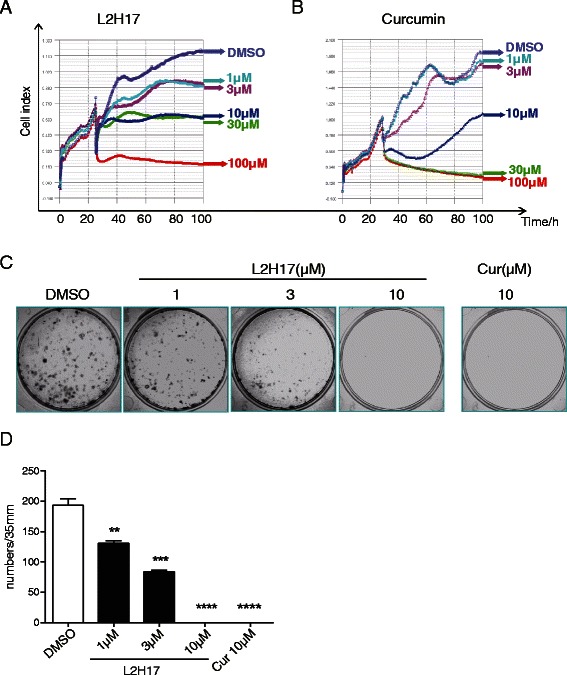
L2H17 suppressed cell growth of CT26.WT cells. After the CT26.WT cells were seeded in E-plates for 24 h, cells were then treated with L2H17 (**a**) or curcumin (**b**) in various concentrations (1, 3, 10, 30 or 100 μM) for 100 h. The cell growth curves were recorded and analyzed by the RT-CES system (*Acea Biosciences Inc., San Diego, CA*). (**c**) Cells were exposed to various concentrations of L2H17 (1, 3, or 10 μM) for 7 days followed by clonogenic assay. Curcumin (10 μM) or DMSO (3 μL) was added as the positive and negative controls, respectively. Visualized colonies were then photographed and calculated. The data were obtained from three independent experiments performed in triplicate. And the representative photos were shown. (**d**) The calculated colonies were presented as mean ± SEM. The indicated differences are significant ** *P* < 0.01, *** *P* < 0.001, and **** *P* < 0.0001, *t*-test, compared to the DMSO-treated group

To further verify the anti-cancer properties of L2H17, the RT-CES system was used to test the inhibitory effect on the proliferation of CT26.WT cells. After the CT26.WT cells were seeded in E-plates for 24 h, cells were then treated with L2H17 or curcumin in various concentrations for 100 h, and the cell growth curves were recorded and analyzed by the RT-CES system. As shown in Fig. 2a, a strong inhibition in cell viability was observed in a dose-dependent manner during 0-76 h after L2H17 treatment. Similar to the result from Fig. 1d, L2H17 exhibited comparative anti-proliferation effect compared to curcumin (Fig. 2b).

Colony formation ability characterizes the independent survival ability and environmental adaptability of cancer cells, and to a certain extent, can reflect the tumorigenicity after tumor cell metastasis. We then investigated the effect of L2H17 on the clonogenic survival ability of CT26.WT cells by plate colony formation assay. As shown in Fig. 2c and 2d, similar to curcumin, L2H17 dose-dependently inhibited the clonogenic survival ability of CT26.WT after seven days of treatment. In summary, we confirm that L2H7 showed potent inhibitory effect on colon cancer cell growth *in vitro* and may be a potential candidate for the treatment of colon cancer.

### L2H17 induced a G0/G1 cell cycle arrest and apoptosis in CT26.WT cells

**Fig. 3:**
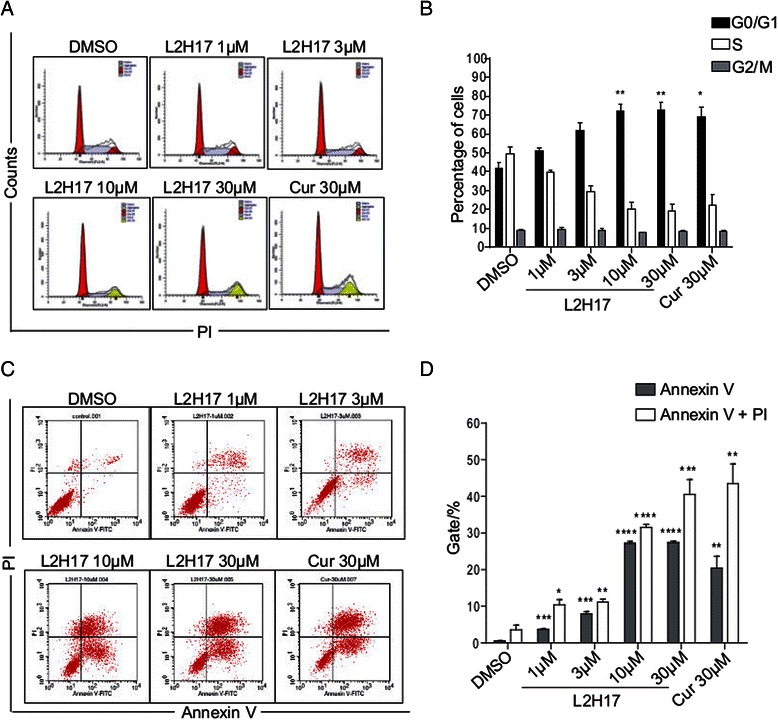
L2H17 induced cell cycle arrest and apoptosis in CT26.WT cells. (**a**) CT26.WT cells were treated with L2H17 (1, 3, 10, or 30 μM) or curcumin (30 μM) for 24 h, and the cell cycle distribution was analyzed by the cytometry (*Becton Dickinson FACSCalibor, BD Biosciences, Franklin Lakes, NJ*). The data were obtained from four independent experiments performed in triplicate. And the representative histogram of the cell cycle distribution was shown. (**b**) The mean of the four values was calculated, and the percentages of cells in Sub-G0/G1, S and G2/M phases are shown. (**c**) The effects of L2H17 and curcumin on the induction of apoptosis in CT26.WT cells. CT26.WT cells were treated with DMSO or the indicated concentration of L2H17 (1, 3, 10, or 30 μM) or curcumin (30 μM) for 48 h, and then stained with Annexin V and PI, followed by detection using flow cytometry. The data were obtained from four independent experiments performed in triplicate. Representative data are shown (**c**), and the percentages of cells with early apoptosis (**d**) are shown. Data are presented as the mean ± SEM. The indicated differences are significant * *P* <0.05, **P < 0.01, ***P < 0.001, and ****P < 0.0001, *t*-test, compared to the DMSO-treated group

Curcumin has been reported to exhibit its anti-cancer effect through its G0/G1 arrest effect [19]. Thus, we also examined the effect of L2H17 treatment on cell cycle progression by flow cytometry. We found that L2H17 induced a dose-dependent cell cycle arrest in the G0/G1 phase of CT26.WT significantly (Fig. 3a & 3b). After treatment of CT26.WT with L2H17 at the concentration of 30 μM for 24 h, G0/G1-phase cells were increased to 72.73 ± 4.125 % (*P* <0.001 v.s. DMSO control), which is comparative to that of curcumin-treated group (69.17 ± 8.541 %).

We also examined whether L2H17 could induce apoptosis in CT26.WT cells. CT26.WT cells were treated with L2H17 (1, 3, 10, or 30 μM) or curcumin (30 μM) for 48 h, and then stained with Annexin V and PI for apoptosis analysis using flow cytometry. As shown in Fig. 3c & 3d, exposure of CT26.WT to L2H17 resulted in a significant dose-dependent increase in early (Annexin V^+^, PI^−^) and late apoptotic cells (Annexin V^+^, PI^+^). At the dose of 10 μM, there was a 48.0-fold (*P* < 0.0001) increase in the population of CT26.WT cells undergoing apoptosis compared to the vehicle control cells (DMSO group). Similar apoptosis index patterns were obtained when CT26.WT cells were exposed to curcumin.

**Fig. 4:**
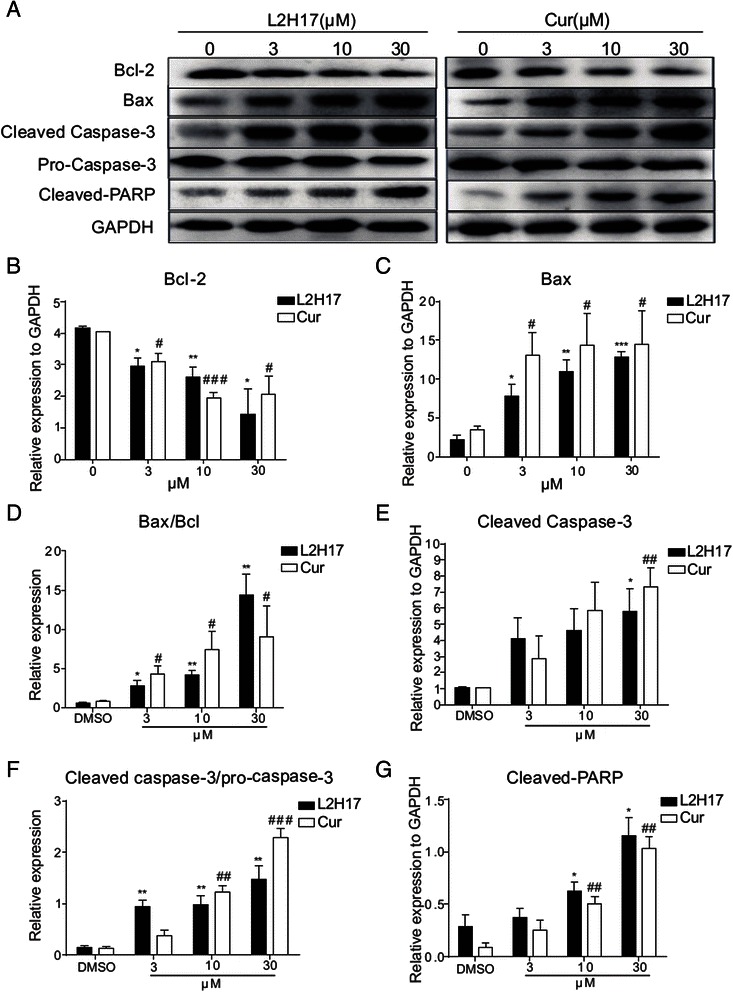
The effects of L2H17 on the expression of apoptosis related proteins in CT26.WT cells. (**a**) CT26.WT cells were incubated with L2H17 (0, 3, 10, or 30 μM) or curcumin (30 μM) for 48 h. The total protein was extracted and the expression of Bcl-2, Bax, caspase-3 and PARP was examined by western blot. GAPDH was shown as the control of equal loading. Data shown are representative of three independent experiments. (**b**-**g**) The histograms of the relative density of Bcl-2 (**b**), Bax (**c**), Bax/Bcl (**d**), Cleaved Caspase-3 (**e**), Cleaved caspase-3/pro-caspase (**f**), and Cleaved PARP (**g**) are shown. The indicated differences are significant * *P* <0.05, ** *P* < 0.01, and *** *P* < 0.001, *t*-test, L2H17-treated compared to the DMSO-treated group; # *P* <0.05, ## *P* < 0.01, and ### *P* < 0.001, *t*-test, curcumin-treated compared to the DMSO-treated group

In order to further confirm the apoptotic induction effects of L2H17, the expression of apoptosis-related proteins was examined using western blot. As shown in Fig. 4a–4c, CT26.WT exposed to L2H17 for 48 h showed a dose-dependent reduction in the level of anti-apoptotic Bcl-2 protein, with a concomitant increase in the level of pro-apoptotic Bax and the Bax/Bcl-2 ratio, compared with the control cells (Fig. 4d). Moreover, the expression of procaspase-3 was clearly decreased, while cleaved caspase-3 increased notably, with a concomitant increased cleaved caspase-3/ procaspase-3 ratio (Fig. 4e & 4f). PARP-1 can be inactivated by caspase cleavage, which is considered to be a marker of apoptosis. As shown in Fig. 4a & 4g, cleaved PARP-1 was induced dose-dependently after L2H17 treatment. Similar results were also found in the curcumin-treated group (Fig. 4). All these data indicated that L2H17 may exert its growth inhibition through cell cycle arrest and apoptosis induction.

### L2H17 decreased cell migration and invasion of CT26.WT cells

**Fig. 5:**
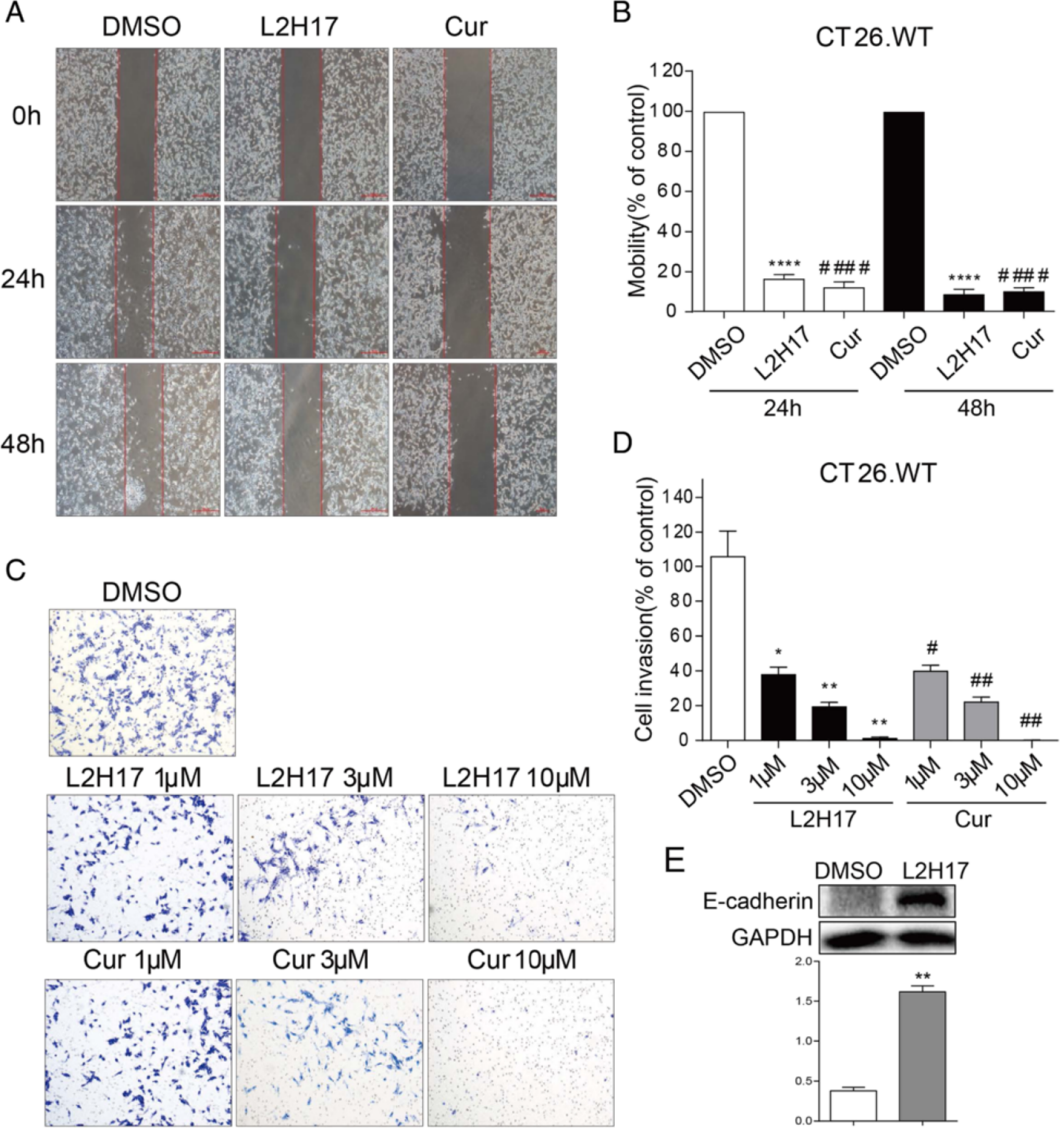
L2H17 inhibited cell migration and invasion of CT26.WT cells. CT26.WT cells were seeded and allowed to adhere overnight. The next day, the “reference line” was drawn at the bottom of each plate. After washed with PBS, the cells were treated with L2H17 (10 μM), curcumin (10 μM) or DMSO (vehicle control) in the presence of mitomycin C for indicated times (24 or 48 h). (**a**) Representative photomicrographs are shown from one out of four independent cell migration experiments. On 0 h images, the “reference line” was drawn to represent the starting points for CT26.WT cells. (**b**) The mobility of CT26.WT cells was calculated for L2H17- or curcumin-treated groups as compared with the DMSO-treated (vehicle control) group for each experiment in different fields. The bar chart represents means (± SEM) of four independent experiments. CT26.WT cells were seeded in the transwell plate that was coated with Matrigel. Then the cells were treated with either L2H17 (1, 3, or 10 μM) or curcumin (1, 3, or 10 μM) for 24 h. And then the invasion cells were fixed, stained and viewed under a light microscope (×100; *Nikon, Tokyo, Japan*). (**c**) Representative photomicrographs of DMSO control (vehicle control), L2H17- and curcumin-treated invading cells are shown from one out of four independent. (**d**) The numbers of invading cells in L2H17- or curcumin-treated groups as compared with the DMSO-treated (vehicle control) group for each experiment in at least 15 representative microscopic fields. (**e**) CT26.WT cells were treated with L2H17 (10 μM) for 12 h. Then the total protein was extracted and the expression of E-cadherin were detected by Western blot analysis. The column figures show the normalized optical density from the data from three independent experiments. The bar chart represents means (± SEM) from one out of three independent experiments. The indicated differences are significant *P <0.05, **P < 0.01, ***P < 0.001, and ****P < 0.0001, *t*-test, L2H17-treated compared to the DMSO-treated group; #*P* <0.05, ##*P* < 0.01, ###*P* < 0.001, and ####*P* < 0.0001, *t*-test, curcumin-treated compared to the DMSO-treated group

To study the inhibitory effects of L2H17 in cell migration, we adopted a scratch wound model in the presence of mitomycin C which inhibited cell proliferation. Wound areas were marked and photographed at 0, 24, and 48 h, respectively. As shown in Fig. 5a and 5b, the mobility of CT26.WT cells decreased to 16.18 % (24 h) and 8.38 % (48 h) in L2H17-treated group (*P* < 0.0001), and 11.82 % (24 h) and 9.84 % (48 h) in curcumin-treated group (*P* < 0.0001), respectively.

We then examined the effect of L2H17 on the invasiveness of CT26.WT cells using the Matrigel invasion assay as described in Methods. As shown in Fig. 5c & 5d, L2H17-treated cells markedly decreased their adhesive ability in a dose-dependent manner compared to the DMSO control group (*P* < 0.05). In addition, curcumin showed similar effects on cell migration and invasion.

E-cadherin, a member of the classic cadherin family, is frequently associated with dedifferentiation and invasion in a variety of human cancers. Loss of E-cadherin in cancer cells is often associated with markers of “epithelial-to-mesenchymal transition” (EMT), including colon cancer cells [20]. In order to figure out the mechanism for the inhibitory effect of L2H17 in colon cancer cell migration and invasion, we measured the expression of E-cadherin in L2H17-treated CT26.WT cells. As shown in Fig. 5e, L2H17 treatment significantly increased the E-cadherin expression level in CT26.WT cells.

### L2H17 inactivated NF-κB signaling of CT26.WT cells

**Fig. 6:**
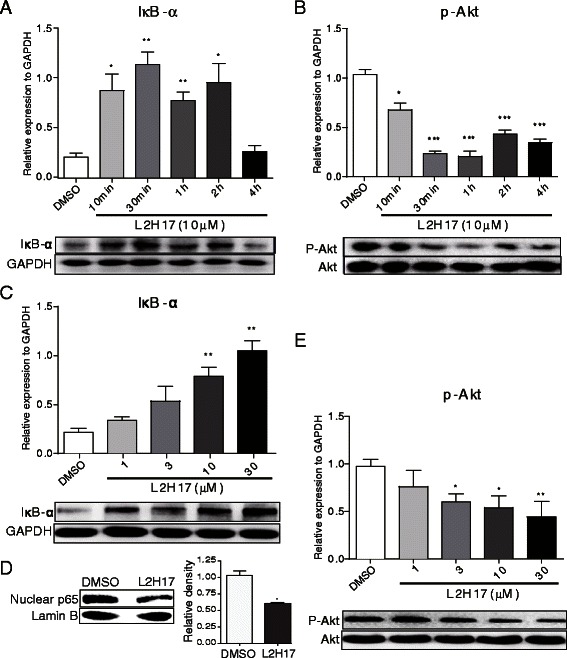
NF-κB and Akt pathways may be involved in the anti-cancer effects of L2H17. CT26.WT cells were treated with L2H17 (10 μM) for indicated times (0, 10, 30, 60, 120, or 240 min, **a** & **b**) or treated with indicated concentrations of L2H17 (1, 3, 10, or 30 μM, **c** & **d**) for 30 min. Then the total protein was extracted and the expression of IκB-α (**a**,**c**), p-Akt (**b**,**e**) proteins were detected by Western blot analysis. (**d**) CT26.WT cells were treated with L2H17 (10 μM) or vehicle control (DMSO) for 1 h. Then the cells were harvested and lysed, nuclear extracts were prepared, and p65 expression levels in the nucleus were measured by Western blot assay. The column figures show the normalized optical density from the data from three independent experiments. Data are presented as the mean ± SEM. The indicated differences are significant * *P* <0.05, ** *P* < 0.01, and *** *P* < 0.001, *t*-test, compared to the DMSO-treated group

A potential role for NF-κB in the initiation, promotion and progression of tumor, including stomach cancer [21], colon cancer [22], pancreatic cancer [23], liver cancer [24], lung cancer [25], has been already evident. The degradation of IκB-α, which is an inhibitory factor of NF-κB, leads to NF-κB activation via promoting p65 translocation from the cytosol to nucleus. In order to explore the possible mechanism through which L2H17 exerts its anti-cancer effect, we examined the degradation of IκB-α after L2H17 treatment. CT26.WT cells were treated with L2H17 (10 μM) for indicated times, or treated with indicated concentration of L2H17 (1, 3, 10, or 30 μM) for 30 min. As shown in Fig. 6a & 6c, L2H17 treatment time-dependently and dose-dependently inhibited the degradation of IκB-α. Also, as evident from the Fig. 6d, L2H17 treatment reduced nuclear p65 protein level, partially demonstrating the potential molecular mechanism underlying the effect of L2H17 on colon cancer is through its inhibition of NF-κB signaling.

Previous studies indicate that PI3K signaling pathways are implicated in cancer invasion [26]. Thus, we then examined whether the invasion-suppressing activity of L2H17 is related to attenuation of the Akt expression using western blot analysis. As shown in Fig. 6b & 6e, L2H17 significantly reduced Akt activation in both a time-dependent and a dose-dependent manner. These data suggest that attenuation of the Akt signaling pathway by L2H17 presumably leads to a reduced level of cell invasion.

### L2H17 possessed anti-tumor activity in vivo

**Fig. 7:**
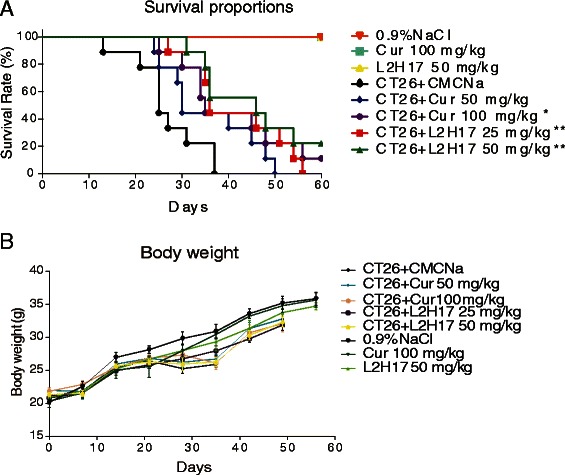
Treatment of L2H17 inhibited the tumorigenesis of CT26.WT cells in BALB/c mice. CT26.WT cells were harvested and injected intravenously into the BALB/c mice (3 × 10^5^ cells/mouse). Simultaneously, the mice were administered orally with either L2H17 (25 mg/kg/day or 50 mg/kg/day), curcumin (50 mg/kg/day, 100 mg/kg/day) or vehicle control (1 % CMC-Na) for 60 days, while the tumor-free control mice were treated with the L2H17 (50 mg/kg/day), curcumin (100 mg/kg/day), or 1 % CMC-Na as the negative control (n = 9 in each group). Shown are survival (**a**) and body weight change (**b**). The indicated differences are significant *P <0.05 and **P < 0.01, *log-rank* test, compared to the CT26.WT + CMC-Na-treated group

Finally, we evaluated the anti-tumor effects of L2H17 in vivo. The mice injected with CT26.WT cells were administered orally with either L2H17 (25 mg/kg/day or 50 mg/kg/day), curcumin (50 mg/kg/day or 100 mg/kg/day) or vehicle (1 % CMC-Na) for 60 days. After injection with 3 × 10^5^ murine CT26.WT cells, all animals developed multiple lung metastases (data not shown). However, as shown in Fig. 7a, treatment of L2H17, at both low and high dosage, significantly extends the survival in the tumor mice (*P* < 0.01). Curcumin exhibited its anti-tumor effects only at the dose of 100 mg/kg/day (*P* < 0.05). Besides, there is no significant differences in survival and body weight change among the non-tumor curcumin (100 mg/kg/day), non-tumor L2H17 (50 mg/kg/day), and non-tumor CMC-Na (1 % CMC-Na/day), which indicating that no significant side effects of curcumin and L2H17 were found at these therapeutic doses (Fig. 7a & 7b).

## Discussion

A lot of literatures have demonstrated that chalcones possess chemopreventive and anti-tumoural activities among several interesting biological properties [27, 28]. Juan Rodrigues et al. have reported the potent effects of new 4-maleamic acid and 4-maleamide peptidyl chalcone derivatives against human prostate cancer *in vitro* and in vivo [29]. It has been also reported that derivatives of hydroxychalcones showed effective cytotoxicity against melanoma cells B16-10 [30]. More recent studies have shown the use of Flavokawain B, a kava chalcone, as a chemotherapeutic and chemopreventive compound [31]. Especially, chalcone derivatives of 2-acetyl thiophene show the cytotoxic and apoptotic effects on human colon cells [32]. Based on the potent pharmacological properties of chalcones, we prepared four synthetic chalcones to investigate their anti-cancer effects on colon cancer cell lines by MTT assay. MTT assay is able to measure cell metabolic activity mostly throughout mitochondria metabolism [33]. Our data show that treatment of these four synthetic chalcone derivatives dose-dependently reduced cell viability in SW620 (Fig. 1b), HCT 116 (Fig. 1c), and CT26.WT (Fig. 1d), with IC_50_ values much lower than those of curcumin. An attractive candidate as an anticancer agent is characterized by a high degree of cancer-selectivity in its cytotoxic effect. In our results, L2H17, with its potent cytotoxic effect on cancerous cells and the weakest effect on noncancerous cells (Fig. 1), was further investigated for its effect on the carcinogenesis of colon cancer. In addition, RT-CES assay and colony formation assay demonstrated that L2H17 presents as good inhibitory effects on colon cancer cell growth as curcumin (Fig. 2).

We then investigated cell apoptosis, cell cycle and the expression of the apoptotic related proteins following L2H17 treatments. Our data showed that L2H17, as well as curcumin, induced prominent cell apoptosis in CT26.WT (Figs. 2 and 3). Aberration of apoptosis has been implicated not only in preventing the development and progression of premalignant colonic epithelial cells to colon tumors but also in resistance of tumors to conventional cancer therapies [34]. In our study, L2H17 and curcumin significantly increase the expression of Bax and suppress the expression of Bcl-2. Interestingly, both agents induced the activation of procaspase-3, which was associated with a significant increase in the cleavage and activation of PARP, a target protein of caspase-3 (Fig. 4e–g). The changes are consistent with the levels of Bcl-2 and Bax expression, supporting the conclusion that the intrinsic pathway is the major mechanism of apoptosis following L2H17 exposure (Fig. 4b–4d). Additionally, the G0/G1 arrest of CT26.WT upon the two compounds treatment partly explained their growth suppression on colon cancer cell (Fig. 3a–3b).

Metastasis, proceeding through several consecutive steps including neovascularization, migration, intravasation, circulation of lymphatic or vascular channels, extravasation and ultimate metastatic colonization is the most malignant feature during cancer progression and accounts for almost 90 % of mortality [35]. Since invasion and migration are a critical event in cancer progression and especially in metastasis, the inhibitory effect of L2H17 on cell invasion and migration was evaluated. Our results showed that L2H17 exhibits similar effects on cell migration and invasion as compared to that of curcumin via increasing the expression of E-cadherin in CT26.WT cells (Fig. 5).

Activated p-Akt, existing in most cancer cells, is a major anti-apoptotic pathway which is frequently hyperactivated in most cancers and is able to promote cell growth and survival by activating its downstream substrates. In addition, activated p-Akt is shown to be associated with various tumor promotion and progression by regulating its downstram pathways such as NF-κB [36]. The activation of NF-κB pathway is a crucial event in tumor growth and progression [37]. In addition to inhibition of apoptosis, activated NF-κB may control cell cycle progression by regulating the expression of important cell cycle regulatory proteins such as cyclin D1 andcyclin dependent kinase 2 (CDK2), further contributing to the tumour growth [38]. Potentiation of cancer cell migration and invasion also explains the role of NF-κB in tumor promotion and progression [38]. In the present study, L2H17 dose-dependently inhibited the degradation of IκB and the phosphorylation of Akt, indicating the inhibition of L2H17 on the activation of NF-κB and Akt pathway, which is consistent with our initial results performed by MTT assay, colony formation assay, flow cytometry and cell migration and invasion assay (Fig. 6).

L2H17 showed some obvious advantages over curcumin, the known potent chemopreventive agent against colon cancer, in the inhibition of conlon cancer cells *in vitro* and in vivo. Despite showing the similar cytotoxic effect on colon cancer cell lines, L2H17 is much more moderate on the noncancerous HL7702 than curcumin (Fig. 1), indicating its low toxicity and exceptional safety. Although there is no significant toxicity in both L2H17-treated and curcumin-treated group in vivo (Fig. 7a), the statistically significant dosage of L2H17 is lower than curcumin, 25 mg/kg/day and 100 mg/kg/day, respectively (Fig. 7).

## Conclusions

Our results show the potent chemopreventive effect and molecular mechanisms of L2H17 on colon cancer both *in vitro* and in vivo, and provide us a promising agent, which is expected to be developed into an effective therapeutic drug against colon cancer. Further experiments are surely needed to explore the direct targets of L2H17 on colon cancer cells.

## References

[1] Azcarate-Peril MA, Sikes M, Bruno-Barcena JM (2011). The intestinal microbiota, gastrointestinal environment and colorectal cancer: a putative role for probiotics in prevention of colorectal cancer?. Am J Physiol Gastrointest Liver Physiol.

[2] Chorost MI, Datta R, Santiago RC, Lee B, Bollman J, Leitman IM (2004). Colon cancer screening: where have we come from and where do we go?. J Surg Oncol.

[3] De Simone V, Franze E, Ronchetti G, Colantoni A, Fantini MC, Di Fusco D, et al. Th17-type cytokines, IL-6 and TNF-alpha synergistically activate STAT3 and NF-kB to promote colorectal cancer cell growth. Oncogene. 2014;34:3493–503. 10.1038/onc.2014.286 PMC4493653 25174402

[4] Fukata M, Shang L, Santaolalla R, Sotolongo J, Pastorini C, Espana C (2011). Constitutive activation of epithelial TLR4 augments inflammatory responses to mucosal injury and drives colitis-associated tumorigenesis. Inflamm Bowel Dis.

[5] Go ML, Wu X, Liu XL (2005). Chalcones: an update on cytotoxic and chemoprotective properties. Curr Med Chem.

[6] Singh P, Anand A, Kumar V (2014). Recent developments in biological activities of chalcones: A mini review. Eur J Med Chem.

[7] Bukhari SN, Jasamai M, Jantan I (2012). Synthesis and biological evaluation of chalcone derivatives (mini review). Mini Rev Med Chem.

[8] Maioral MF, Gaspar PC, Rosa Souza GR, Mascarello A, Chiaradia LD, Licinio MA (2013). Apoptotic events induced by synthetic naphthylchalcones in human acute leukemia cell lines. Biochimie.

[9] Jamier V, Marut W, Valente S, Chereau C, Chouzenoux S, Nicco C (2014). Chalcone-Coumarin derivatives as potential anti-cancer drugs: an in vitro and in vivo investigation. Anticancer Agents Med Chem.

[10] Drutovic D, Chripkova M, Pilatova M, Kruzliak P, Perjesi P, Sarissky M (2014). Benzylidenetetralones, cyclic chalcone analogues, induce cell cycle arrest and apoptosis in HCT116 colorectal cancer cells. Tumour Biol.

[11] Wu CM, Lin KW, Teng CH, Huang AM, Chen YC, Yen MH (2014). Chalcone derivatives inhibit human platelet aggregation and inhibit growth in human bladder cancer cells. Biol Pharm Bull.

[12] Zhou XW, Ma HL, Zhang X, Jing SY, Miao JY, Zhao BX (2014). Synthesis of 6-cinnamoyl-2H-benzo[b][1,4]oxazin-3(4H)-ones and their effects on A549 lung cancer cell growth. Eur J Med Chem.

[13] Dimmock JR, Elias DW, Beazely MA, Kandepu NM (1999). Bioactivities of chalcones. Curr Med Chem.

[14] Wu J, Li J, Cai Y, Pan Y, Ye F, Zhang Y (2011). Evaluation and discovery of novel synthetic chalcone derivatives as anti-inflammatory agents. J Med Chem.

[15] Ostan R, Lanzarini C, Pini E, Scurti M, Vianello D, Bertarelli C (2015). Inflammaging and Cancer: A Challenge for the Mediterranean Diet. Nutrients.

[16] Ramamoorthi G, Sivalingam N (2014). Molecular mechanism of TGF-beta signaling pathway in colon carcinogenesis and status of curcumin as chemopreventive strategy. Tumour Biol.

[17] Baskaran R, Madheswaran T, Sundaramoorthy P, Kim HM, Yoo BK (2014). Entrapment of curcumin into monoolein-based liquid crystalline nanoparticle dispersion for enhancement of stability and anticancer activity. Int J Nanomedicine.

[18] Kim TD, Fuchs JR, Schwartz E, Abdelhamid D, Etter J, Berry WL (2014). Pro-growth role of the JMJD2C histone demethylase in HCT-116 colon cancer cells and identification of curcuminoids as JMJD2 inhibitors. Am J Transl Res.

[19] Mayol L, Serri C, Menale C, Crispi S, Piccolo MT, Mita L (2015). Curcumin loaded PLGA-poloxamer blend nanoparticles induce cell cycle arrest in mesothelioma cells. Eur J Pharm Biopharm.

[20] Huels DJ, Ridgway RA, Radulescu S, Leushacke M, Campbell AD, Biswas S (2015). E-cadherin can limit the transforming properties of activating beta-catenin mutations. EMBO J.

[21] Suarez G, Romero-Gallo J, Piazuelo MB, Wang G, Maier RJ, Forsberg LS (2015). Modification of Helicobacter pylori Peptidoglycan Enhances NOD1 Activation and Promotes Cancer of the Stomach. Cancer Res.

[22] Simoes AE, Pereira DM, Gomes SE, Brito H, Carvalho T, French A (2015). Aberrant MEK5/ERK5 signalling contributes to human colon cancer progression via NF-kappaB activation. Cell Death Dis.

[23] Shi M, He X, Wei W, Wang J, Zhang T, Shen X (2015). Tenascin-C induces resistance to apoptosis in pancreatic cancer cell through activation of ERK/NF-kappaB pathway. Apoptosis.

[24] Zhen Y, Pan W, Hu F, Wu H, Feng J, Zhang Y (2015). Exogenous hydrogen sulfide exerts proliferation/anti-apoptosis/angiogenesis/migration effects via amplifying the activation of NF-kappaB pathway in PLC/PRF/5 hepatoma cells. Int J Oncol.

[25] Birsu Cincin Z, Unlu M, Kiran B, Sinem Bireller E, Baran Y, Cakmakoglu B (2015). Anti-proliferative, apoptotic and signal transduction effects of hesperidin in non-small cell lung cancer cells. Cell Oncol (Dordr).

[26] Tsai HR, Yang LM, Tsai WJ, Chiou WF (2004). Andrographolide acts through inhibition of ERK1/2 and Akt phosphorylation to suppress chemotactic migration. Eur J Pharmacol.

[27] Nowakowska Z (2007). A review of anti-infective and anti-inflammatory chalcones. Eur J Med Chem.

[28] Lin YM, Zhou Y, Flavin MT, Zhou LM, Nie W, Chen FC (2002). Chalcones and flavonoids as anti-tuberculosis agents. Bioorg Med Chem.

[29] Hengartner MO (2000). The biochemistry of apoptosis. Nature.

[30] Navarini AL, Chiaradia LD, Mascarello A, Fritzen M, Nunes RJ, Yunes RA (2009). Hydroxychalcones induce apoptosis in B16-F10 melanoma cells via GSH and ATP depletion. Eur J Med Chem.

[31] Ji T, Lin C, Krill LS, Eskander R, Guo Y, Zi X (2013). Flavokawain B, a kava chalcone, inhibits growth of human osteosarcoma cells through G2/M cell cycle arrest and apoptosis. Mol Cancer.

[32] de Vasconcelos A, Campos VF, Nedel F, Seixas FK, Dellagostin OA, Smith KR (2013). Cytotoxic and apoptotic effects of chalcone derivatives of 2-acetyl thiophene on human colon adenocarcinoma cells. Cell Biochem Funct.

[33] Mosmann T (1983). Rapid colorimetric assay for cellular growth and survival: application to proliferation and cytotoxicity assays. J Immunol Methods.

[34] Swamy MV, Patlolla JM, Steele VE, Kopelovich L, Reddy BS, Rao CV (2006). Chemoprevention of familial adenomatous polyposis by low doses of atorvastatin and celecoxib given individually and in combination to APCMin mice. Cancer Res.

[35] Yoon SO, Park SJ, Yun CH, Chung AS (2003). Roles of matrix metalloproteinases in tumor metastasis and angiogenesis. J Biochem Mol Biol.

[36] Toker A, Yoeli-Lerner M (2006). Akt signaling and cancer: surviving but not moving on. Cancer Res.

[37] Karin M, Greten FR (2005). NF-kappaB: linking inflammation and immunity to cancer development and progression. Nat Rev Immunol.

[38] Jung YJ, Isaacs JS, Lee S, Trepel J, Neckers L (2003). IL-1beta-mediated up-regulation of HIF-1alpha via an NFkappaB/COX-2 pathway identifies HIF-1 as a critical link between inflammation and oncogenesis. FASEB J.

